# Biocircuits in plants and eukaryotic algae

**DOI:** 10.3389/fpls.2022.982959

**Published:** 2022-09-23

**Authors:** Mayna da Silveira Gomide, Matheus de Castro Leitão, Cíntia Marques Coelho

**Affiliations:** ^1^Laboratory of Synthetic Biology, Department of Genetics and Morphology, Institute of Biological Science, University of Brasília (UnB), Brasília, Distrito Federal, Brazil; ^2^School of Medicine, Federal University of Juiz de Fora (UFJF), Juiz de Fora, Minas Gerais, Brazil

**Keywords:** biocircuits, plants, eukaryotic algae, synthetic chromosomes, synthetic biology

## Abstract

As one of synthetic biology’s foundations, biocircuits are a strategy of genetic parts assembling to recognize a signal and to produce a desirable output to interfere with a biological function. In this review, we revisited the progress in the biocircuits technology basis and its mandatory elements, such as the characterization and assembly of functional parts. Furthermore, for a successful implementation, the transcriptional control systems are a relevant point, and the computational tools help to predict the best combinations among the biological parts planned to be used to achieve the desirable phenotype. However, many challenges are involved in delivering and stabilizing the synthetic structures. Some research experiences, such as the golden crops, biosensors, and artificial photosynthetic structures, can indicate the positive and limiting aspects of the practice. Finally, we envision that the modulatory structural feature and the possibility of finer gene regulation through biocircuits can contribute to the complex design of synthetic chromosomes aiming to develop plants and algae with new or improved functions.

## Introduction

Photosynthetic organisms, such as plants and algae, have been discussed as promising candidates to overcome the existing challenges in the different areas of bioeconomy, food and feed, environment, and health. However, efficient methods for multiple genetic and metabolic engineering are essential to access this potential. In this sense, synthetic biology tools could contribute towards this goal.

Genetic circuits combine, in a network manner, genetic parts in several switches that are system units able to perceive an input, process the information and generate an output (Andres et al., 2019). Endogenous biocircuits have been known since the 60s, once Monod and Jacob recognized the resemblance of the gene expression control in the lactose and tryptophan operons with electric circuits (Monod and Jacob, 1961). Nonetheless, it was not until the 2000s that scientists developed the first synthetic genetic circuits (Elowitz and Leibler, 2000; Gardner et al., 2000). Since then, this synthetic biology strategy has been used to produce photosynthetic organisms, such as plants and algae, with desirable traits (Rasala et al., 2012; de Lange et al., 2018).

Earlier than synthetic biocircuits, the first construct with a centromere, an origin of replication, a selectable marker, and telomeres was transformed and maintained in yeast (Murray and Szostak, 1983). From then on, artificial chromosomes have been proposed as a platform for introducing genes of interest to developing organisms with new or improved functions. In plants, minichromosomes, engineered through telomere-mediated truncation (Yu et al., 2006, 2007), can be envisioned as the ground foundation for synthetic chromosome development in these organisms (Birchler, 2015). In this regard, these synthetic structures could be discussed as a safe landing platform to introduce genes finely regulated through synthetic circuits to obtain plants and algae expressing intended phenotypes.

**Figure 1:**
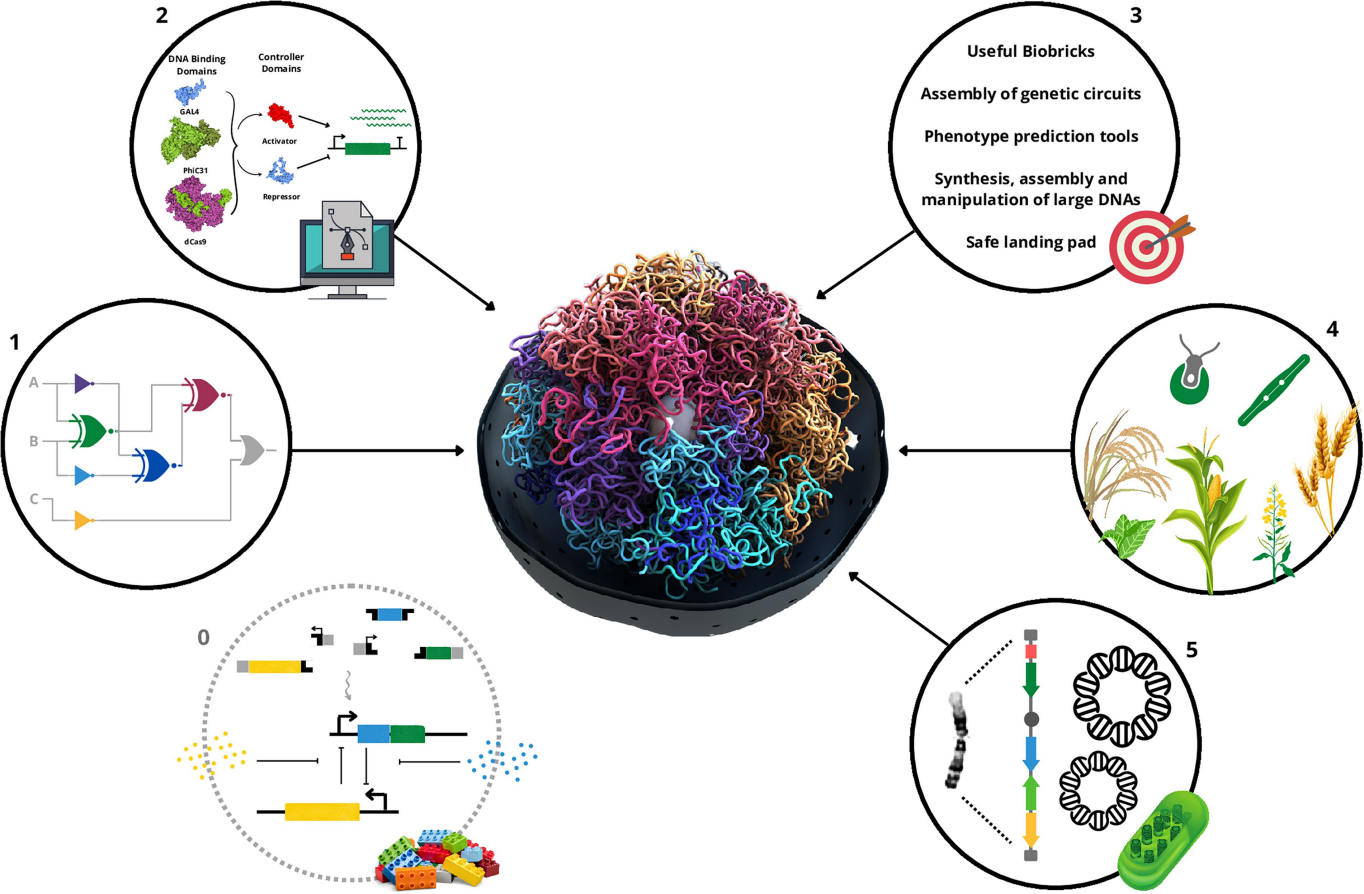
Schematic summary of the important steps, challenges, achievements, and prospective usage in synthetic chromosomes for the biocircuits in plants and algae. First, a repository of reliable, functional, orthogonal, scalable biological parts (0) is essential to the assembly of biocircuits (1). Also, tools that allow fine control of the expression of genes that compose the biocircuits with computational methods that can use the biological parts to assemble genetic circuits and predict the phenotype from the genotype are very welcome (2). Those together with the need of a repository for usable biological parts, tools that allow synthesis, assembly and manipulation of large DNAs, and safe harbors for the genetic circuits represent the main challenges in this area (3). Nevertheless, high vitamin A Golden crops, biosensors, and trans-kingdom genetic circuit plants, in addition to biofabric algae have been developed (4). As a perspective, biocircuits could greatly contribute to the control of gene expression once artificial/synthetic chromosomes become the landing platform for plants and algae development with improved or new functions (5). The 3D design of the molecules in the number 2 scheme was generated by Illustrate (Goodsell et al., 2019).

In this review, we will focus on presenting a brief history of biocircuits, the requirements and challenges for their widespread use, and the achievements completed in plants and eukaryotic algae (Figure 1). We will also discuss the perspective of this synthetic biology tool to provide a more precise gene expression control system for artificial chromosomes and how all these advances will contribute to the development of functions of interest (Figure 1).

## Genetic circuits foundation and development

After Jacob and Monod proposed that biological elements are connected in regulatory circuits (Monod and Jacob, 1961), other researchers extended these ideas. In this process, an expansion was to evaluate genes as on–off devices, linking the genetic control expression to Boolean algebra functions (Kauffman, 1969; Thomas, 1973; Sugita, 1975). In Boolean terms, the expression of a gene can be seen as binary variables, assuming 1 when the gene is expressed and 0 when it is not. This proposal is valuable for the design of genetic logic gates whose outputs obey their specific truth tables (Wieland and Fussenegger, 2012; Miyamoto et al., 2013).

Emerging from the theoretical studies, the first synthetic circuit was the genetic toggle switch planned to work as a binary on–off gene state in *Escherichia coli* (Gardner et al., 2000). This system was composed of two inducible promoters controlling the expression of each other’s repressor protein, being one of the genes cistronically followed by the coding sequence of the reporter green fluorescence protein (GFP). Thus, an oscillatory reporter expression was expected after coordinated induction. In the same year, Elowitz and Leibler also designed and constructed a synthetic oscillatory network using repressor proteins (Elowitz and Leibler, 2000), with a similar rationale and extended complexity. The model works as an extension of the first prototypical oscillator, the Goodwin oscillator (Goodwin, 1965). This system consists of elements that regulate themselves as a negative feedback loop. However, this repressilator exhibited undesirable noise behavior and, as the toggle switch, stability was affected by stochastic features of the regulatory components.

As the complexity of synthetic circuits increases, the functional description of biological parts and standardization of assembly methods could help to minimize unpredicted outcomes and facilitate genetic constructions. The Registry of Standard Biological Parts (RSBP), created for this purpose (Endy, 2005; Cameron et al., 2014), catalogs and stores biological genetic parts, such as promoters, terminators, and protein-coding sequences. The standardized BioBrick format allows an assembly approach based on low-frequency restriction enzymes of *E. coli* (Knight, 2003). Currently, the International Genetically Engineered Machine (iGEM) Foundation is responsible for the public record of these parts. The institution also promotes a student competition for synthetic biology development with projects that have a significant source of approaches to test genetic circuits in several organisms, including plants and algae.

Since 2010, the iGEM teams that aimed at plant studies have been developing projects of biosensors, bioremediation, expression of proteins of interest or compounds with pharmaceutical properties, and CRISPRs systems. Due to the technical challenges, time-consuming required to work with plants, and the competition’s scheduled time, most projects are not fully accomplished. However, it is achievable to highlight substantial contributions to the synthetic biology of plants. Among these are the toolkit of parts available and the perception structures characterized, like some inducible promoters or those responsive to stress conditions. An extensive repository of these and other parts is available.^1^

Gibson assembly (Gibson et al., 2009), founded on PCR-overlap, and Golden Gate assembly (Engler et al., 2008, 2009), founded on type IIS restriction enzymes, are other proposed methodologies, based on one tube reaction. The Golden Gate approach has received considerable interest in plant synthetic biology for providing the joining of several parts at once into a receptor plasmid with relative simplicity. This cloning technique has evolved into modular assemblies enabling to engineer multiple transcriptional units and consists of the aims of MoClo (Engler et al., 2014) and GoldenBraid (Sarrion-Perdigones et al., 2011) strategies. The dissemination of these techniques raised once again the need to standardize the parts used. Thus, researchers defined, in a collective effort, standards for fusion sites of genetic parts for cloning and assembly methods using type IIs enzymes. This guidance, called Common Syntax for eukaryotic parts assembly (Patron et al., 2015), was adopted by iGEM for the so-designated phytobricks. The MoClo and Golden Braid groups developed specific toolkits for plants, which are presented in Table 1. The DBTL (design, build, test and learn) methodology used to establish these kits provides a range of parts that can be easily changed in a construction and contributes to modules’ functionality information for the future development of plant circuits.

**Table 1:** Synthetic biology tools for plants and algae.

Goal		Name/platform	Brief description	Reference
Parts andassembly toolkits	Collections	MoClo plant parts kits/Addgene (https://www.addgene.org/kits/patron-moclo/)	Kit collection with 95 Golden Gate standardizedbiological parts for plant transformation.	Engler et al., 2014
		MoClo plant parts II and infrastructure kit/Addgene (https://www.addgene.org/kits/stuttmann-moclo-plant-infrastructure/)	Kit collection with 95 multigenic modules compatible with MoClo system for plant constructs, including vectors connecting MoClo and Gateway platforms and vectors for yeast two hybrid assays and bacterium-to-plant translocation.	Gantner et al., 2018
		Nicola Patron lab plasmids/Addgene (https://www.addgene.org/Nicola_Patron/)	It holds more than 200 plasmids, deposited by the Nicola Patron lab, to be distributed to the scientific community, in addition to the MoClo plant parts kits.	Multiple references mentioned in Addgene table.
		MoClo CRISPR/Cas toolkit for Plants (https://www.addgene.org/kits/nekrasov-moclo-plant-crispr/)	Kit collection with 95 plasmids for transformation and expression of CRISPR/Cas nucleases, base editors, gRNA backbones, and promoters in plants.	Hahn et al., 2020
		pENFRUIT gateway collection (https://www.addgene.org/browse/article/2397/)	The collection comprises several functional DNA parts (fruit promoters, GOIs and terminators) ready to be assembled in a combinatorial way using Gateway system.	Estornell et al., 2009
		Golden Braid 4.0 (https://gbcloning.upv.es/)	A platform that encompasses several tools for modular assembly of multigenic DNA structure to be used in Plant and Fungal synthetic biology applications. It holds GB parts collection, experimental data and tools to convert a DNA sequence into a GB element, to do *in silico* simulation of DNA assembly reactions, and a CRISPR tool for gene editing and gene regulation.	Sarrion-Perdigones et al., 2013; Vazquez-Vilar et al., 2017
	iGEM initiatives	Concordia – Clean green lipid machines:synthetic biology tools for microalgaeproject/ iGEM (http://2014.igem.org/Team:Concordia/Project/Microalgae)	Concordia developed a collection of compatible and non-compatible BioBrick parts, such as promoters with varying strentghs, terminators, fluorescent proteins, localization tags, antibiotic markers, and CRISPR/Cas. *Chlorella vulgaris*, *Chlorella kessleri* and *Chlamydomonas reinhardtii* were the chassis organisms.	–
		Humboldt Berlin – ChlamyHUB project/iGEM (https://2019.igem.org/Team:Humboldt_Berlin)	ChlamyHub established a toolkit of MoClo and no MoClo parts, including promoters, terminators, secretion signals, reporters and markers, and vectors to engineer *Chlamydomonas reinhardtii*’s genome. Additionally, some MoClo parts were genes of interest.	–
		Marbug – Phaectory project/iGEM (https://2013.igem.org/Team:Marburg)	Phaectory made the *Phaeodactylum tricornutum* accessible to synthetic biology, providing a kit of biological parts that include resistance genes, inducible promoters, terminators, different signal peptides for protein localization, and Hepatitis B antibody heavy and light chain coding sequences. Continuity: OpenPlast project (https://2021.igem.org/Team:Marburg”), that aimed to characterize chloroplast parts in a cell free system and share automation and software tools.	–
Computational instruments		Open plant (https://www.openplant.org/)	It is an initiative that aims to implement open technologies and practices for plant synthetic biology.	–
		Synthetic biology open language (SBOL) (https://sbolstandard.org/)	Standardization of biological pieces representation to facilitate constructions and exchange of synthetic biology designs.	Bartley et al., (2015), McLaughlin et al., 2020
		iBioSim (https://geneticlogiclab.org/ibiosim.github.io/)	Algorithm built on the directed acyclic graph (DAG) based mapping techniques used to select parts of digital circuit designs, introducing digital logic from electronic design automation (EAD) to automated genetic design. Continuity: workflow that extends the iBioSim tools to support asynchronous sequential circuit	Roehner and Myers (2014); Nguyen et al., (2019)
		OptCircuit	Design platform whose aim is to construct and fine-tuning biological circuits.	Dasika and Maranas (2008)
		SynBioSS designer	Web-based tool that generates synthetic circuits by using BioBricks parts.	Weeding et al., 2010
		BioPartsBuilder	Software tool that allows building large-scale synthetic pathways from standardized, reusable, biological parts based on Golden Gate assembly.	Yang et al., 2016
		Cello (https://www.cidarlab.org/cello)	Design of complex genetic circuits based on Boolean logic gates connecting transcriptional gates in layers, so the output from one gate serves as input to the next. Continuity: Cello 2.0 (https://zenodo.org/record/4676314#.Yrpm4nbMK5c); and a workflow that extends the Cello tools to support asynchronous sequential circuits.	Nielsen et al., 2016, Jones et al., 2022, Nguyen et al., 2019
Biocircuits	Plants	Golden crops	High carotenoid content plants. Carotenoid is a precursor of vitamin A, whose deficiency leads to blindness and increases infectious disease.	Paine et al., 2005; Diretto et al., 2007; Zhu et al., 2008; Wang et al., 2014; Shumskaya and Wurtzel (2013)
		TNT biosensor	In the presence of TNT, plants’ leaves become whitish by the de-greening circuit.	Antunes et al., 2006; Antunes et al., 2011
		Human pathogenic bacteria biosensor	Plant protoplasts are responsive to the presence of bacterial pathogens.	Czarnecka et al., 2012
		Transkingdom circuit	Plants’ rhizopine production capable of controlling gene expression in rhizosphere bacteria.	Geddes et al., 2019; Haskett et al., 2022
		Cyber-spinach	Development of artificial chloroplasts capable of carbon compound formation after light exposition.	Miller et al., 2020; Scheffen et al., 2021
		Boolean logic gates circuits	Plant systems with gene expression output complying with truth table inputs	Khan et al., 2022 – preprint, Lloyd et al., 2022; Brophy et al., 2022 – preprint
	Algae	Xylanase production	Optimization of xylanase synthesis in *Chlamydomonas reinhardtii.*	Rasala et al., 2012
		Isoprenoids production	Improvement of bisabolene synthesis in *Chlamydomonas reinhardtii* and geraniol synthesis in *Phaeodactylum tricornutum.*	Wichmann et al., 2018; Fabris et al., 2020; George et al., 2020

The genetic network construction tests are mainly conducted in plant chassis, such as *Nicotiana tabacum*, *Nicotiana benthamiana* and *Arabidopsis thaliana*, especially by transient assays performed using leaves agroinfiltration and BY-2 cells (tobacco) or protoplasts transformation. However, the moss *Physcomitrium patens* and the liverwort *Marchantia polymorpha* have also emerged as synthetic biology investigation chassis. Those organisms have completely sequenced genomes, a short life cycle, set laboratory cultivation, and good heterologous protein production. These advantageous features have allowed the specification of some genetic parts and toolkits (Delmans et al., 2017; Reski et al., 2018; Sauret-Güeto et al., 2020).

For a long time, eukaryotic microalgae have been arousing interest regarding their physiology, photosynthetic metabolism, and biotechnological applications, such as biofuels production. Although algae research has been developing molecular tools, the poor expression of heterologous genes from its nuclear genome is a relevant limitation towards this goal, considering the model organism *Chlamydomonas reinhardtii* (Scaife and Smith, 2016). However, some strategies are overcoming these hurdles: use of specific promoters, mutated strains with altered chromatin condensation, intron sequences, subcellular targeting, adoption of more efficient transformation methodologies, and codon optimization (Lauersen et al., 2015; Scaife and Smith, 2016; Vavitsas et al., 2019). The green alga *C. reinhardtii,* as well as the diatom *Phaeodactylum tricornutum*, have been the most explored species as chassis organisms. In this sense, some research groups have developed toolkits aiming to improve the parts’ availability and characterization efforts (Siaut et al., 2007; Scaife and Smith, 2016; Crozet et al., 2018; Butler et al., 2020). Also, as an example, iGEM groups that developed such toolkits are presented at Table 1.

## Circuit design and control

The design of a synthetic genetic circuit demands fulfilling several criteria. Although the synthesis of short DNA sequences has become cheaper and more accessible, making it easier for the modular assembly of parts, there are still limitations, such as the maximum fragment size sustained by a plasmid and the incapability of synthesizing high-quality long DNA fragments. There are also drawbacks to the delivery and manipulation of these long sequences and a lack of knowledge of epigenetic chromosomal interactions and mechanisms that control the genome’s three-dimensional structure (Ostrov et al., 2019). Additionally, there are specific needs for synthetic circuit design, as the engineered circuits’ ability to function and integrate into biological systems in a predictable manner, but not suffering undesired endogenous interference or *vice-versa*. Therefore, synthetic circuits need to use fully characterized biological parts that are independent, reliable, orthogonal, tunable, composable and scalable (Lucks et al., 2008). In this sense, part sequences usually come from other organisms or are artificially designed as hybrid sequences.

Essential elements for creating more effective genetic circuits are precise and efficient gene control expression systems. For plants, following these premises, chemically inducible promoters have been applied using mechanisms from *E. coli* tetracycline-regulated de-repression, *Aspergillus nidulans* ethanol induction, and systems of animal steroid receptors activation (Gatz and Quail, 1988; Schena et al., 1991; Caddick et al., 1998).

Furthermore, the search for new transcriptional controllers has guided the design of synthetic regulators. Some research groups fused DNA-binding domains, such as the yeast GAL4 or the bacterium LexA, with the transactivating domain of the herpes viral protein VP16 or with *A. thaliana* repressor domains. In some cases, a steroid receptor was fused to control the system induction or repression (Weinmann et al., 1994; Aoyama and Chua, 1997; Zuo et al., 2000; Schaumberg et al., 2016). Additionally, an expanded library of synthetic promoters with variable strengths using a range of cis and trans-regulatory elements to control a plant’s minimal promoter expression was built (Belcher et al., 2020). The *Neurospora crassa* Q-system was also used as transcriptional gene control. The synthetic structure adjusted three components of the original cluster, a transcriptional activator, a repressor, and the inducing molecule quinic acid (Persad et al., 2020). Already in the GB3.0 system (Vazquez-Vilar et al., 2017), the authors developed two transcription control mechanisms. The first uses phiC31 integrase fused to activation (Gal4 or VP64) or repression (Arabidopsis BRD) domains, and the second is based on transcriptional modules from flavonoids biosynthesis, using the Rosea 1 and Delila regulators.

It is worth mentioning the development of optogenetically regulated controllers for plants: the red light responsive split transcriptional system, based on phytochrome B (PhyB) and phytochrome-interacting factor 6 (PIF6) connection (Müller et al., 2014); and the green-light sensitive *Thermus thermophilus* CarH-CarO (transcription factor-operator) dependent on the stability of coenzyme AdoB12 (Chatelle et al., 2018). Yet, recently, promoters controlled by hormone-activated Cas9-based repressors (HACRs) in response to three plant hormones: auxin, gibberellin, and jasmonate (Khakhar et al., 2018), that unlocks the system by degron disruption, were developed. Besides this, synthetic promoters were built based on dCas9: VP64 and specific gRNAs, with their binding sites positioned upstream of a minimal promoter. It was validated by *N. benthamiana* infiltration and *A. thaliana* transgenic plants. The system used an inducible promoter ethylene-responsive to drive gRNAs expression and verified the transcriptional controller’s orthogonality in a multiplex test (Kar et al., 2022).

It is important to note that the biological parts described in some synthetic transcriptional complexes do not comply with all criteria of the biological circuits design principles because they depend on endogenous components. Nevertheless, the above systems allow the genetic toggle switch construction in which genes can exist in two stable states switching from one to another and interacting to form genetic circuits. Post-transcriptional and translational control systems, mainly RNA-based tools, such as RNA interference, microRNAs, ribozymes, or aptamers, were also developed. A comprehensive list of these tools is described elsewhere (Andres et al., 2019).

Another approach to control gene expression is using site-specific recombinases and placing their recognition sites flanking target parts to be rotated. For instance, a recent study showed the functionality of six serine integrases (Ints) to perform the 180° rotation of coding and promoter sequences of the designed genetic switches, thereby controlling the GFP reporter expression (Gomide et al., 2020). A further study used the phiC31 integrase and its cognate protein, the recombination directionality factor (RDF), to switch between activated or deactivated states of the reporter genes by inversion of regulatory parts in *N. benthamiana* (Bernabé-Orts et al., 2020).

All the above mentioned expression control systems open up uncountable combinatorial possibilities for the genetic circuits designed for plants. One can also oversee that agricultural applications are under interest to activate synthetic genetic networks with identified promoters responsive to biotic and abiotic stress (Singhal et al., 2016; Muthusamy et al., 2017) or synthesized to switch on with fertilization (Crawford et al., 2010).

On the other hand, there was a delay in algae tools development that allow for controlled nuclear gene expression. In *C. reinhardtii*, until recently, successful results had not been reached, despite testing several endogenous, chimeric, and viral promoters for gene expression (Wang et al., 2012). Nevertheless, some studies could improve transcriptional control, such as the fusion of the HSP70A-RBCS2 promoters that increased endogenous and exogenous nuclear gene expression levels (Schroda et al., 2000). Additionally, Scranton and collaborators identified a range of cis-motifs in highly expressed nuclear genes from *C. reinhhardtii* and generated a set of novel functional synthetic algal promoters (Scranton et al., 2016). Inducible and repressible promoters were also available. Among them, it is worth listing: the metal-responsive CYC6 promoter, induced by nickel and repressed by copper ions (Ferrante et al., 2008); the METE promoter, repressible by vitamin B12 (Helliwell et al., 2014); the sulfur starvation-induced promoter of LHCBM9 (Sawyer et al., 2015); an light-inducible promoter from *Dunaliella* sp. (Baek et al., 2016); the salt-inducible promoter from *C.reinhardtii* GPDH3 gene (Beltran-Aguilar et al., 2019); an alcohol-inducible promoter from *A. nidulans* (Lee et al., 2018); and the promoters inducible by digoxin and β-estradiol, that were previously effective for other eukaryotic organisms and adapted for *P. tricornutum* (Kassaw et al., 2022).

Navarro and Baulcombe extended the gene expression control models for *C. reinhardtii* using fluorescent models to characterize miRNAs for a post-transcriptional switching-off regulation approach (Navarro and Baulcombe, 2019). In that same algae, Mehrshahi and collaborators described another RNA-based tool for controlling gene expression (Mehrshahi et al., 2020). From the endogenous THI4, they developed novel riboswitches that respond to different ligands and can be used to design synthetic genetic circuits (Mehrshahi et al., 2020). Even though the essential toolkits available for synthetic research advancement in algae are still lagging compared to plants, these works represent substantial steps in this direction.

Computational prediction tools that allow the automated design of the synthetic circuits are primordial for plants and algae, particularly advancing from genotype to phenotype prediction. The availability of characterized biological parts, standard assembly approaches, collections of parts, and improvement of testing systems enabled the conception of computer-aided tools. Nevertheless, it must consider the limitations of plant and algae engineering regarding the efficiency and time-consuming of the existing transformation methods. The synthetic circuit tests in simple model chassis also require caution, especially in plants, since these systems’ behavior might not be the same between the testing platforms and the final organism. With all these considerations, some tools and databases are available for the choice and assembly assistance of the experimental genetic parts. More information about these platforms is in Table 1.

## Examples of engineered genetic circuits and their biotechnological applications

Over the years, the expansion of synthetic biologic toolkits has allowed the construction of some genetic circuits in plants that generate predictable outputs after input processing in a network manner (Table 1). Considering that the ultimate goal in these organisms is crop improvement, some of the examples of engineered genetic circuits will focus on them. Until now, most of the synthetic genetic circuits developed in crops relate to synthetic metabolic pathways. Some of these pathways aimed to increase carotenoid content, the vitamin A precursors (de Lange et al., 2018). The deficiency of this vitamin results in blindness and increases infectious diseases being a prominent concern in parts of the developing world. These systems had, as input signals, plant and bacterial encoding enzyme genes (phytoene synthase, phytoene desaturase, carotenoid desaturase, lycopene β-cyclase, and/or β-carotene ketolase) presented in several combinatorial manners. Therefore, this approach could overcome the bottlenecks of the carotenoid biosynthesis network in crops such as rice (Paine et al., 2005), potato (Diretto et al., 2007), maize (Zhu et al., 2008), and wheat (Wang et al., 2014), creating high-content carotenoid plants, so-called golden crops. Nonetheless, after years of carotenoid pathway studies, some information on this multi-enzyme complex system still lacks a better understanding of improved metabolic engineering circuit design (Shumskaya and Wurtzel, 2013). Likewise, consumer acceptance of those engineered crops needs to be addressed, as the Golden Rice still awaits further exploration (de Lange et al., 2018).

Concerning examples of complex genetic circuits for plants, there is still a handful of those described in the literature. One of the first circuits fully designed was built to be a plant biosensor capable of detecting 2,4,6-trinitrotoluene (TNT; Antunes et al., 2011). This plant detection system was based on bacterial chemotactic components adapted for plants. The periplasmic binding protein (PBP) was redesigned to recognize TNT as a ligand in the plant cell apoplast. Once connection occurs, the protein remodeling allows the linkage with the chimeric transmembrane transduction signaling protein, Trg-PhoR, whose activation induces the PhoB-VP64. This transcription factor has an affinity for the synthetic promoter PlantPho, which controls a response signal that promotes the loss of the leaves’ green color. The named de-greening circuit was detailed elsewhere (Antunes et al., 2006) and consists of genes that inhibit chlorophyll synthesis and initiate its degradation. Although this work was an important example of complex plant-engineered genetic circuits with biotechnological applications, there were drawbacks and further adjustments will be needed for this plant system to be used as means of detection of contaminants, explosives, or other chemical agents. Soon after, following the same rationale of using plants as biosensors, Czarnecka and collaborators developed a mechanism for plants to detect human bacterial pathogens (Czarnecka et al., 2012). In this work, the strategy relied on a biological genetic switch, based on a transcriptional autofeedback loop. The input signal was the bacterial flagellin that allowed plant protoplasts to amplify their endogenous defense response to pathogens, which would ultimately lead to an output signal of a deteriorated plant, inhibiting its commercialization.

Yet, there were two outstanding advancements in plant synthetic biology research. The first one was the establishment of a transkingdom genetic circuit of plant-dependent synthesis and signaling of rhizopine to control bacteria in the rhizosphere (Geddes et al., 2019). Rhizopines are rare molecules in nature synthesized by a few rhizobia species in legume nodules during N_2_-fixing symbiosis. This groundbreaking work creates plant control possibilities for specific soil microbiota members to perform essential tasks for crop improvement, such as Nitrogen (N_2_) fixation and nutrient solubilization. N_2_ fixation has been a central concern because the chemical nitrogenous fertilizers, which constituted the base for the green revolution, are a limited resource. Its use also results in high water pollution levels and the eutrophication of lakes and rivers (Bano and Sheikh, 2016). The second exciting work was the development of the so-called cyber-spinach. In this study, the researchers used microfluidics to obtain an artificial chloroplast by encapsulating, operating spinach photosynthetic membranes, and combining it with an improved laboratory-designed enzyme pathway, the CETCH cycle version 7.0 (Miller et al., 2020). The light inputs drive the reactions network for the CO_2_ conversion, leading to the multicarbon compound glycolate as output. This artificial photosynthetic pathway is more efficient than the natural one having potential biotechnological applications. Despite this, several questions remain, such as the system compatibility with the living cell machinery, its long-time lifespan, and the scalability of an economical operation. Focusing on both techniques’ progression, their respective research groups have been improving some of the biological components of those systems (Scheffen et al., 2021; Haskett et al., 2022). Nevertheless, these works can be viewed as the founder stone for several research applications.

Notably, thorough genetic biocircuits following the Boolean logic gates concepts have just been accomplished for plants. Based on CRISPRi (interference), the assembled gates inhibit the transcription initiation using a dCas9 guided by sgRNAs to a target promoter (Khan et al., 2022 – preprint). The second system has constant output signals by circuits working under recombinases control instead of the transitory condition in the previous work. This research used the recombinases Flp, Cre, and B3 to control a luciferase output by excising the promoter, coding, or terminator sequence. Thus, it obeys Boolean logic gates control in Arabidopsis protoplasts or roots from transgenic plants (Lloyd et al., 2022). Another observed progress was for a result with direct interference in plant development. The work tested synthetic transcription regulators compounding Boolean genetic gates to activate or repress gene expression. Significantly, some of the circuits could successfully control root development in *A. thaliana* transgenic plants (Brophy et al., 2022 – preprint).

In algae, the advancements of synthetic biology toolkits are paving the way for studies aiming to produce important chemical molecules in these organisms (Table 1). Exemplifying, Rasala and collaborators showed that xylanase, an important industrial enzyme, could achieve a relevant augment in *C. reinhardtii* cell lysates. In this study, *C. reinhardtii xyn1* gene expression was coupled with the virus 2A self-cleavage peptide and with an antibiotic selection gene (Rasala et al., 2012). Likewise, as described above for plants, in algae, the synthetic genetic circuits initially developed relate to synthetic metabolic pathways. For decades, it has been an effort toward metabolic engineering of algae to produce chemically diverse isoprenoid molecules due to their importance in medicine, agriculture, cosmetics, biofuels and several other applications (Wang et al., 2012; Wichmann et al., 2018; Butler et al., 2020). Wichmann and collaborators combined a heterologous overexpression of bisabolene synthase genes and repression of squalene synthase gene expression through microRNA, leading to improvement of bisabolene productivity, which is the sesquiterpene biodiesel precursor (Wichmann et al., 2018). However, the authors argue that the capacity for bisabolene production is still below the industrial demands, and improvement in genetic transformation tools is still needed. Further, *P. tricornutum* has a raised potential as a candidate for terpenoid heterologous synthesis. The photosynthetic background of enzymes and precursors possibly favors the synthesis of these compounds. Thus, geraniol was synthesized in this diatom microalgae and high productivity was studied between genome insertion and episomal expression (Fabris et al., 2020; George et al., 2020).

## Perspectives in circuit design to gene regulation in plants and algae artificial chromosomes

Before discussing how genetic circuits could contribute to the control of gene expression in artificial chromosomes, it is essential to define them and to report advancements in this area in plant and eukaryotic algae. Artificial chromosomes are chemically synthesized DNA molecules with a centromere, origins of replication, telomeric regions, and other regulatory parts, mimicking a natural chromosome’s behavior (Murray and Szostak, 1983). Initially aiming to investigate the structural requirements of natural chromosomes within the cell cycle, artificial chromosomes currently have numerous functions, promises, and challenges. It is worth highlighting their importance in genetic essentiality studies (Hutchison et al., 2016) and for constituting safe harbors for targeting transgenes (Kuroiwa et al., 2000; Gaeta et al., 2013). Additionally to being, in the future, the basis for highly adjustable and specialized chassis organisms creation for the biotechnology industry. The first independent artificial chromosomes were constructed in the unicellular organisms *Saccharomyces cerevisiae* (Murray and Szostak, 1983) and *E. coli* (O’Connor et al., 1989), known as Yeast Artificial Chromosomes (YACs) and Bacterial Artificial Chromosomes (BACs), respectively. They played a critical role in the eukaryotic genome sequencing and characterization, including in the Human Genome Project (Lander et al., 2001; Venter et al., 2001). With the advancement of DNA synthesis technology and the improvement of genomics, groups and consortia have assembled synthetic chromosomes and genomes. They are similar or reduced to their natural equivalent and capable of replacing them, as in the case of *Mycoplasma* (Gibson et al., 2010; Hutchison et al., 2016), *S. cerevisiae* (Annaluru et al., 2014; Richardson et al., 2017), and *E. coli* (Fredens et al., 2019; Kurokawa and Ying, 2019). In multicellular organisms, the recent advances are in artificial minichromosome approaches, such as those used in human cells, Human Artificial Chromosomes (HACs; Farr et al., 1995; Harrington et al., 1997; Ikeno et al., 1998), and plant, Plant Artificial chromosomes (PACs; Yu et al., 2006, 2007).

Plants and eukaryotic algae are underrepresented in advances in this area. The formation and function of plant centromeres still present significant challenges that limit bottom-up approaches (Carlson et al., 2007; Ananiev et al., 2009), such as the complex epigenetic influence and species-specific sequence repetitions (Han et al., 2006, 2018; Birchler and Han, 2009). Using top-down methods, the telomere-mediated truncation technique (Farr et al., 1992), for instance, has already been used to create artificial minichromosomes in several cultivars and models such as maize (Yu et al., 2006, 2007), rice (Xu et al., 2012), barley (Kapusi et al., 2012), wheat (Yuan et al., 2017), *A. thaliana* (Nelson et al., 2011; Teo et al., 2011; Murata et al., 2013), and *Brassica napus* (Yan et al., 2017). In algae, advances are even timider, consisting mainly of the phases that precede the creation of synthetic chromosomes. Highlights include the assembly of a few genomic chromosomes or the genome of algal organelles in host organisms, such as the two *P. tricornutum* chromosomes assembled in *S. cerevisiae* (Karas et al., 2013), the assembly of the chloroplast genome of the green alga *C. reinhardtii* in yeast and transformed into *C. reinhardtii* (O’Neill et al., 2012), and the synthetic mitochondrial genome of *Thalassiosira pseudonana* cloned in yeast and *E. coli* (Cochrane et al., 2020), respectively.

Artificial chromosomes can be ideal Synthetic Biology tools, working as safe harbors for exogenous gene insertion that compose biosynthetic pathways of molecules of interest. Furthermore, the building of new genetic circuits could contribute to refining their expression control. However, it will be necessary to overcome challenges, such as the manipulation difficulties of large DNA fragments (Ostrov et al., 2019) and the low meiotic transmission rate in some organisms (Han et al., 2007; Masonbrink et al., 2012), to establish these chromosomes as a robust approach.

## Discussion

As strategies from Synthetic Biology advance, the use of biocircuits to optimize plants and algae for diverse purposes is obviously aimed, and, indeed, substantial advancements have already been achieved. However, intrinsic functional variation of biological compounds often results in synthetic systems performing below expectations. Thus, most researchers still prefer widely used biological parts with well-established functionalities, for example, some constitutive promoters, such as CaMV35S. Nevertheless, such components have limitations on their use in more expanded and regulated arrangements. The functional consolidation of the new parts deposited in current databases by the next generation of research will be fundamental for more effective biocircuits achievements in these species.

Noticeably, technical issues still hamper studies of systems designed for plants and algae considering other model organisms. For example, Cello, a software developed and employed to design 45 successful genetic circuits in *E. coli,* was also recently used by Chen and coworkers to build functional biocircuits in yeast, taking advantage of the overwhelming knowledge available to these organisms (Nielsen et al., 2016; Chen et al., 2020). Additionally, transient assays are a more straightforward way to test the operability of circuit components and get rapid answers. Nonetheless, even for this approach type, different from microorganisms or organisms with established cell lines, the time for sampled organisms’ preparation is slower and the transformant phenotypes usually exhibit a wide range of variation. Besides, the adaptation needed for analytical protocols and executions is time-consuming. Kits, facility services, and high throughput methods are also less available. Thus, transposing the built system to stable insertion in a final interest organism represents additional difficulties, including the cost–benefit assessment for the investment in the technology.

Many of the genetic constructions desired for plants and algae demand improvement in the knowledge regarding carbon and photosynthetic metabolism. Likewise, it is necessary a better comprehension of the genome organization and its relationship with the exogenous DNA sequences integrated to compound the biocircuit. Finally, understanding the functional relationships in metabolic networks of sequenced genes is still needed in many pathways.

All these challenges emphasize the importance of establishing new genetic parts and regulatory constructs capable of functioning satisfactorily not only in the model chassis but also in the final target organisms. Computational tools can, therefore, make an important contribution to this challenging expansion of viable biological components for construction of functional new genetic circuits. Furthermore, a perspective that also could contribute to mitigating stochastic effects in the biocircuits is direct the designed constructions to genomic safe harbors. Besides a better knowledge of possible safe harbors in the endogenous chromosomes, the synthetic chromosomes are promising structures to insert complex networks to be expressed, once challenges of stabilization and meiotic division are overcome.

## Author contributions

CMC and MSG conceptualized the mini-review. MSG, CMC and MCL designed the table. MCL designed the figure. All the authors contributed to the writing and the final revision of this manuscript.

## Conflict of interest

The authors declare that the research was conducted in the absence of any commercial or financial relationships that could be construed as a potential conflict of interest.

## Publisher’s note

All claims expressed in this article are solely those of the authors and do not necessarily represent those of their affiliated organizations, or those of the publisher, the editors and the reviewers. Any product that may be evaluated in this article, or claim that may be made by its manufacturer, is not guaranteed or endorsed by the publisher.

## Footnotes

^1^ https://parts.igem.org/Collections/Plants
